# Cannabis use increases surgical, medical, and psychosocial complications after lower extremity fracture fixation and shows compounded risk with concurrent nicotine use

**DOI:** 10.1038/s41598-026-55842-w

**Published:** 2026-06-03

**Authors:** Christopher D. Hamad, Michelle Nwufo, Joshua Wiener, Timothy Liu, Thomas E. Olson, Paul Walker, Soroush Shahamatdar, Nora A. Galoustian, Nick Yusin, Autreen Golzar, David C. Kaelber, Nicholas M. Bernthal, Christopher Lee, William L. Sheppard

**Affiliations:** 1 Department of Orthopaedic Surgery, University of California, Los Angeles, CA USA; 2 David Geffen School of Medicine at UCLA, 1225 15th Street – Suite 3144B, Santa Monica, Los Angeles, CA 90404 USA; 3 Case Western Reserve University School of Medicine, Cleveland, OH USA; 4 University of Southern California, Los Angeles, CA USA; 5 Population and Quantitative Health Sciences, Case Western Reserve University and the Center for Clinical Informatics Research and Education, The MetroHealth System, Cleveland, OH USA

**Keywords:** Cannabis, Nicotine, Lower extremity fractures, Orthopaedic trauma, Postoperative complications, Propensity score matching, Diseases, Health care, Medical research, Risk factors

## Abstract

Cannabis use is rapidly increasing in the United States, yet its perioperative implications remain poorly understood, particularly in trauma populations. Despite expanding legalization, the perioperative effects of cannabis on surgical outcomes remain uncertain. This retrospective cohort study used electronic health record data to evaluate the association between cannabis use, with and without concurrent nicotine exposure, and postoperative outcomes following orthopedic lower extremity trauma fixation. We conducted a retrospective cohort study using the TriNetX Research Network, a global federated electronic health record platform that aggregates de-identified clinical data from more than 90 health systems. Adult patients who underwent surgical fixation of lower extremity fractures between 2015 and 2023 were stratified into four cohorts: cannabis only users (n = 2421), nicotine only users (n = 36,966), concurrent users (n = 2338), and non-users. Primary outcomes included surgical and medical postoperative complications; secondary outcomes included psychosocial outcomes and coagulation parameters (PT, aPTT). Binary outcomes were evaluated using absolute risk differences, risk ratios (RR), and 95% confidence intervals (CIs). Continuous variables were compared using independent samples t-tests assuming unequal variances. Cannabis-only users demonstrated higher rates of deep implant infection, nonunion or malunion, reoperation, transfusion, readmission, anxiety, and depression compared with matched non-users. Nicotine-only users showed higher rates of wound complications, infection, nonunion or malunion, reoperation, transfusion, pneumonia, myocardial infarction, death, opioid use, chronic pain, readmission, anxiety, and depression compared with matched non-users. Concurrent cannabis-and-nicotine users had higher rates of superficial wound infection, deep implant infection, reoperation, amputation, transfusion, pneumonia, respiratory failure, opioid use, chronic pain, readmission, and anxiety compared with matched cannabis-only users. Coagulation measures were not consistently different across exposure groups. Cannabis use was associated with surgical and psychosocial complications in this large retrospective cohort. Nicotine use was associated with broader adverse effects, and concurrent cannabis-and-nicotine exposure was associated with compounded perioperative risk. These findings support further prospective evaluation of perioperative substance use screening and counseling strategies in surgical populations. Prospective studies quantifying cannabis exposure are needed to define dose–response effects and mechanisms.

**Supplementary Information:**

The online version contains supplementary material available at 10.1038/s41598-026-55842-w.

## Introduction

Cannabis use has increased rapidly across the United States, with per capita consumption rising more than 15-fold since 1992^1^. Since 2022, the number of daily cannabis users has surpassed that of daily alcohol users, with nearly half of cannabis consumers reporting daily or near-daily use^1^. As of 2021, approximately 19% of U.S. adults reported cannabis use, with the highest prevalence among young adults, a demographic frequently represented in orthopedic trauma populations^2–4^. As of 2025, recreational cannabis is legal in 24 states and Washington, D.C., with several others considering active legislation^5^. With expanding legalization and normalization of use, national exposure rates are expected to continue rising in the coming years.

Cannabis is now widely accessible, and its growing acceptance has outpaced our understanding of its perioperative and systemic safety. Evaluating cannabis-related health outcomes in large clinical populations is increasingly important as its use becomes common among surgical patients.

Cannabis contains bioactive cannabinoids, primarily delta-9-tetrahydrocannabinol (THC) and cannabidiol (CBD), which act on CB1 and CB2 receptors expressed throughout the cardiovascular, neurologic, musculoskeletal, and immune systems^6,7^. These receptor-mediated effects influence vascular tone, inflammation, wound repair, and immune regulation, which may alter postoperative recovery and complication risk.

Despite widespread legalization, the impact of cannabis exposure on perioperative and surgical outcomes remains uncertain. Prior studies have produced conflicting results, with some linking cannabis use to increased infection and delayed healing, and others reporting no measurable effect. Some reports associate cannabis use with surgical site infection, delayed healing, and altered coagulation profiles, potentially increasing thromboembolic and cardiovascular risk^8–10^. Others have found no such associations, highlighting the need for larger, more representative analyses^11^. Concurrent nicotine use has also been implicated as a compounding factor, though the extent of its interaction with cannabis remains poorly defined^12^. The lack of high-quality, population-level evidence on these interactions has limited clinicians’ ability to counsel patients on perioperative risk and recovery expectations.

This knowledge gap limits the ability of orthopedic surgeons to counsel patients and optimize perioperative care. This study represents one of the largest retrospective cohort analyses examining the independent and combined associations of cannabis and nicotine use with postoperative and psychosocial outcomes following surgical fixation for lower extremity fractures.

## Methods

### Data source and study design

We conducted a retrospective cohort study using the TriNetX Research Network (Cambridge, MA; www.trinetx.com), a federated electronic health record platform that aggregates de-identified clinical data from more than ninety health systems and one hundred thirty million patients worldwide. The platform provides real time access to structured clinical data, including demographics, diagnoses, procedures, medications, and laboratory test results. TriNetX applies internal data quality controls that include schema validation, duplicate suppression, and temporal consistency checks to ensure analytic reproducibility across institutions. Data access was supported by the National Center for Advancing Translational Sciences grant UM1TR004528. The Research Database was accessed on 08/02/2025 and final statistical analyses were completed on 08/12/2025.

All TriNetX data are de-identified in accordance with the Health Insurance Portability and Accountability Act Privacy Rule (45 CFR §164.514[b][1]) using expert-determined methods such as data obfuscation and minimum cell sizes of ten or more individuals per value. Therefore, the dataset does not contain protected health information and is exempt from both HIPAA and institutional review board oversight.

### Cohort selection

**Fig. 1:**
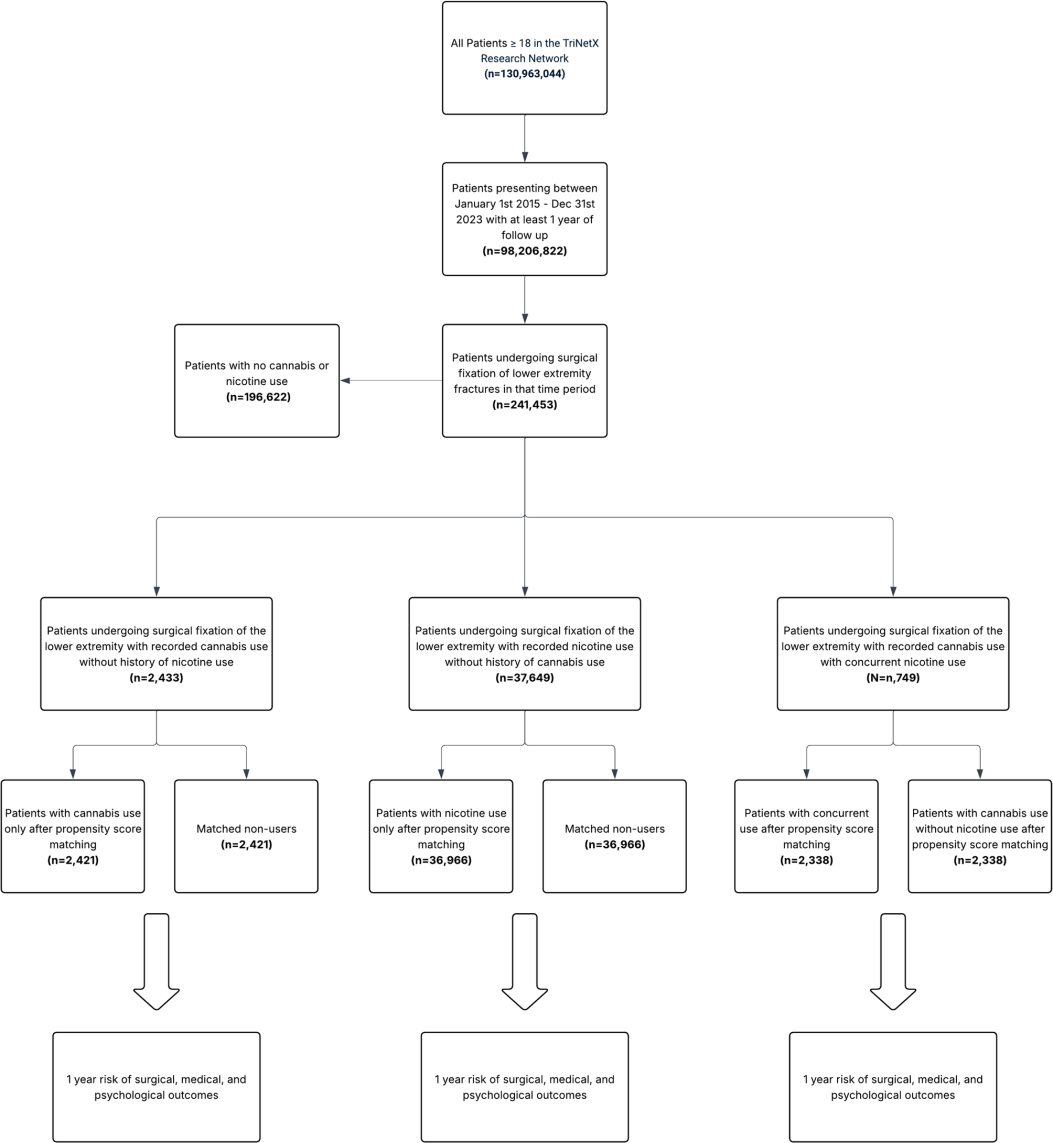
Cohort selection diagram.

Adults aged eighteen years or older who underwent surgical fixation of a lower extremity fracture between January 2015 and December 2023 were eligible for inclusion. This study interval was selected to ensure adequate ICD-10-CM classification and incorporate at least a full year of follow-up. The index event was defined as the date of the first qualifying lower extremity surgery within the study window. Exposure cohorts were defined using preoperative ICD-10-CM documentation of cannabis-related disorders or nicotine dependence. Cannabis exposure was identified using F12 codes, and nicotine exposure was identified using F17 codes; specific subcodes are listed in Supplemental Table 1. Patients were categorized as cannabis-only users, nicotine-only users, concurrent users, or non-users without documented cannabis or nicotine use. These diagnosis codes reflect documented clinical diagnoses rather than direct measures of cannabis or nicotine use behavior. Each cohort was used to generate matched pairs for independent comparisons to assess the association of cannabis and nicotine exposure with postoperative outcomes. Cannabis-only and nicotine-only cohorts were each compared with matched non-users to estimate the associations of each exposure relative to patients without documented cannabis or nicotine use. Concurrent users were compared with matched cannabis-only users to estimate the incremental association of nicotine exposure among patients with documented cannabis use. Therefore, the concurrent-user analysis should be interpreted as the additional risk associated with concurrent nicotine use among cannabis users, rather than as a comparison with substance non-users. Figure 1 demonstrates the stepwise cohort creation process for subsequent analyses.

### Index event and follow-up

The index event for each patient was the first recorded date of lower extremity fracture surgery during the study period. Follow-up began one day after the index event and continued for 365 days. Patients were censored at their last available encounter if follow-up ended before one year. For each outcome assessed, patients with preexisting documentation of that outcome prior to the index event were excluded from the respective analysis to ensure evaluation of incident postoperative events. Laboratory and diagnostic outcomes were assigned based on the earliest qualifying code or result after surgery.

### Outcomes

Primary and secondary outcomes included both medical and surgical complications commonly reported following lower extremity trauma surgery. Outcomes were defined using CPT and ICD-10 codes mapped through TriNetX’s Unified Medical Language System (UMLS) ontology. Events of interest included wound dehiscence, superficial wound infection, deep implant infection, nonunion or malunion, nerve palsy, reoperation, amputation, pneumonia, nosocomial infection, acute respiratory distress syndrome (ARDS), respiratory failure, deep vein thrombosis (DVT), pulmonary embolism (PE), transfusion, myocardial infarction (MI), acute kidney injury, stroke, mortality, all-cause 1 year readmission, anxiety, depression, opioid use, and chronic pain. Laboratory endpoints included prothrombin time (PT) and activated partial thromboplastin time (aPTT).

### Statistical analysis

Propensity score matching (PSM) was applied to reduce baseline confounding between exposure groups. A 1:1 greedy nearest-neighbor algorithm with a caliper of 0.10 pooled standard deviations was employed for each pairwise comparison. Matching variables included age at index, sex, race, BMI, HbA1c, osteoporosis (with and without pathological fracture), atherosclerotic cardiovascular disease, chronic kidney disease, liver disease, COPD, peripheral arterial disease, polysubstance abuse (opioid related disorders, alcoholic related disorders, and other psychoactive substance related disorders), polytraumatized patients, preoperative antibiotics, fracture location, open versus closed fracture types, as well as type of fixation procedure. Additionally, we included all procedure codes used in initial cohort creation as covariates to minimize confounding based on surgical approach. Covariate balance was assessed using standardized mean differences (SMD < 0.10). Specific codes used for both cohort creation and for propensity score matching can be found in Supplemental Table 1.

**Table 1:** Pre and post matching characteristics of cannabis only users, nicotine only users, and concurrent users.

	Pre-match: cannabis-only users					Pre-match: nicotine-only users					Pre-match: concurrent users				
	Cannabis only users (N = 2433)		Non-users (N = 196,622)		SMD	Nicotine only users (N = 37,649 )		Non-users (N = 196,622)		SMD	Concurrent Users (N = 4749 )		Cannabis only users (N = 2433)		SMD
	N, Mean	%, SD	N, Mean	%, SD		N, Mean	%, SD	N, Mean	%, SD		N, Mean	%, SD	N, Mean	%, SD	
Age, mean (SD)	36.289	16.307	53.022	24.711	0.799	49.653	17.47	53.022	24.711	0.157	39.158	14.295	36.289	16.307	0.187
Male, %	1569	64.49%	81,592	41.50%	0.473	25,582	67.95%	141,023	71.72%	0.235	3134	65.99%	1569	64.49%	0.032
Race (White)	1311	53.88%	141,024	71.72%	0.376	20,005	53.14%	81,591	41.50%	0.082	2488	52.39%	1311	53.88%	0.03
BMI, mean (SD)	27.761	6.721	28.486	7.249	0.104	27.962	7.061	28.486	7.249	0.073	26.932	6.709	27.761	6.721	0.124
18–25 kg/m2	740	30.42%	51,279	26.08%	0.096	12,022	31.93%	51,279	26.08%	0.129	1930	40.64%	740	30.42%	0.215
25–30 kg/m2	718	29.51%	56,127	28.55%	0.021	12,289	32.64%	56,127	28.55%	0.089	1651	34.77%	718	29.51%	0.113
30–35 kg/m2	450	18.50%	39,011	19.84%	0.034	8257	21.93%	39,011	19.84%	0.052	994	20.93%	450	18.50%	0.061
35–40 kg/m2	237	9.74%	21,176	10.77%	0.034	4224	11.22%	21,176	10.77%	0.014	499	10.51%	237	9.74%	0.025
40–60 kg/m2	144	5.92%	13,146	6.69%	0.032	2486	6.60%	13,146	6.69%	0.003	283	5.96%	144	5.92%	0.002
HbA1c, mean (SD)	6.031	1.841	6.341	1.781	0.172	6.411	2.055	6.341	1.781	0.036	6.166	2.162	6.031	1.841	0.068
4–5.7%	235	9.66%	21,512	10.94%	0.042	4501	11.96%	21,512	10.94%	0.032	672	14.15%	235	9.66%	0.139
5.7–6.5%	123	5.06%	19,576	9.96%	0.187	3560	9.46%	19,576	9.96%	0.017	318	6.70%	123	5.06%	0.07
6.5–8%	61	2.51%	12,236	6.22%	0.183	2054	5.46%	12,236	6.22%	0.033	154	3.24%	61	2.51%	0.044
8–10%	65	2.67%	9149	4.65%	0.106	1962	5.21%	9149	4.65%	0.026	180	3.79%	65	2.67%	0.063
*Comorbidities*															
Hyperlipidemia	256	10.52%	49,089	24.97%	0.385	8766	23.28%	49,089	24.97%	0.039	630	13.27%	256	10.52%	0.085
Cerebrovascular diseases	141	5.80%	19,303	9.82%	0.150	3894	10.34%	19,303	9.82%	0.018	393	8.28%	141	5.80%	0.097
Diabetes mellitus	203	8.34%	30,640	15.58%	0.225	5654	15.02%	30,640	15.58%	0.016	466	9.81%	203	8.34%	0.051
CAD	117	4.81%	21,225	10.80%	0.225	4410	11.71%	21,225	10.80%	0.029	338	7.12%	117	4.81%	0.098
Other diseases of liver	100	4.11%	8909	4.53%	0.021	2496	6.63%	8909	4.53%	0.092	352	7.41%	100	4.11%	0.142
CKD	105	4.32%	20,234	10.29%	0.231	2871	7.63%	20,234	10.29%	0.093	234	4.93%	105	4.32%	0.029
Osteoporosis	87	3.58%	21,684	11.03%	0.289	2765	7.34%	21,684	11.03%	0.128	138	2.91%	87	3.58%	0.038
COPD	80	3.29%	10,568	5.38%	0.103	6692	17.78%	10,568	5.38%	0.395	521	10.97%	80	3.29%	0.302
PAD	47	1.93%	7988	4.06%	0.125	2343	6.22%	7988	4.06%	0.098	140	2.95%	47	1.93%	0.066
Polytrauma	159	6.54%	7304	3.72%	0.128	2193	5.83%	7304	3.72%	0.099	500	10.53%	159	6.54%	0.143

	Post-match: cannabis-only users					Post-match: nicotine-only users					Post-match: concurrent users				
	Cannabis only users (N = 2421)		Non-users (N = 2421)		SMD	Nicotine only users (N = 36,966)		Non-users (N = 36,966)		SMD	Concurrent users (N = 2338)		Cannabis only users (N = 2338)		SMD
	N, Mean	%, SD	N, Mean	%, SD		N, Mean	%, SD	N, Mean	%, SD		N, Mean	%, SD	N, Mean	%, SD	
Age, mean (SD)	36.318	16.321	36.167	19.538	0.008	49.479	17.506	50.669	22.428	0.059	36.225	13.52	36.27	16.273	0.003
Male, %	1562	64.52%	1565	64.64%	0.003	25,089	67.87%	25,362	68.61%	0.014	1518	64.93%	1509	64.54%	0.008
Race (White)	1305	53.90%	1278	52.79%	0.022	19,538	52.85%	19,791	53.54%	0.016	1232	52.70%	1248	53.38%	0.014
BMI, mean (SD)	27.765	6.69	28.272	7.053	0.074	27.994	7.074	28.397	6.963	0.057	27.403	6.819	27.717	6.613	0.047
18–25 kg/m2	735	30.36%	699	28.87%	0.033	11,598	31.38%	11,419	30.89%	0.011	736	31.48%	716	30.62%	0.019
25–30 kg/m2	715	29.53%	711	29.37%	0.004	11,903	32.20%	12,296	33.26%	0.023	722	30.88%	703	30.07%	0.018
30–35 kg/m2	447	18.46%	440	18.17%	0.008	7999	21.64%	8335	22.55%	0.022	434	18.56%	441	18.86%	0.008
35–40 kg/m2	236	9.75%	241	9.96%	0.007	4090	11.06%	4334	11.72%	0.021	219	9.37%	226	9.67%	0.010
40–60 kg/m2	142	5.87%	149	6.15%	0.012	2434	6.58%	2490	6.74%	0.006	127	5.43%	133	5.69%	0.011
HbA1c, mean (SD)	6.034	1.843	6.333	2.24	0.146	6.41	2.05	6.372	1.912	0.019	6.235	2.227	6.026	1.856	0.102
4–5.7%	234	9.67%	234	9.67%	0.000	4307	11.65%	4228	11.44%	0.007	227	9.71%	231	9.88%	0.006
5.7–6.5%	123	5.08%	111	4.59%	0.023	3436	9.30%	3374	9.13%	0.006	117	5.00%	117	5.00%	0.000
6.5–8%	61	2.52%	40	1.65%	0.061	2006	5.43%	1943	5.26%	0.008	63	2.70%	58	2.48%	0.014
8–10%	65	2.69%	61	2.52%	0.010	1885	5.10%	1833	4.96%	0.006	75	3.21%	63	2.70%	0.030
*Comorbidities*															
Hyperlipidemia	255	10.53%	212	8.76%	0.060	8474	22.92%	8305	22.47%	0.011	249	10.65%	241	10.31%	0.011
Cerebrovascular diseases	138	5.70%	123	5.08%	0.027	3737	10.11%	3627	9.81%	0.010	129	5.52%	133	5.69%	0.007
Diabetes mellitus	203	8.39%	179	7.39%	0.037	5477	14.82%	5412	14.64%	0.005	191	8.17%	194	8.30%	0.005
CAD	117	4.83%	104	4.30%	0.026	4227	11.44%	4201	11.36%	0.002	116	4.96%	114	4.88%	0.004
Other diseases of liver	98	4.05%	90	3.72%	0.017	2295	6.21%	2292	6.20%	0.000	98	4.19%	98	4.19%	0.000
CKD	105	4.34%	88	3.64%	0.036	2837	7.68%	2741	7.42%	0.010	109	4.66%	102	4.36%	0.014
Osteoporosis	87	3.59%	60	2.48%	0.065	2733	7.39%	2564	6.94%	0.018	74	3.17%	69	2.95%	0.012
COPD	79	3.26%	83	3.43%	0.009	6062	16.40%	6068	16.42%	0.000	83	3.55%	80	3.42%	0.007
PAD	47	1.94%	39	1.61%	0.025	2164	5.85%	2105	5.69%	0.007	48	2.05%	44	1.88%	0.012
Polytrauma	156	6.44%	185	7.64%	0.047	2064	5.58%	2083	5.64%	0.002	143	6.12%	156	6.67%	0.023

For binary outcomes, absolute risk differences, risk ratios (RR) and 95% confidence intervals (CIs) were calculated. Kaplan–Meier survival analysis with log-rank testing was used for time-to-event data, and hazard ratios (HR) were derived from Cox proportional hazards models. Continuous outcomes, including PT and aPTT, were compared using independent samples t-tests assuming unequal variances. Outcomes were excluded from analysis if present prior to the index date, unless specifically included per protocol. We considered a *P* value of less than 0.05 to be statistically significant. All statistical analyses were performed within the TriNetX Analytics environment using validated, platform-embedded algorithms to maintain data security and reproducibility.

### Ethics and data use

This study was conducted using de-identified data from the TriNetX Research Network and was performed in accordance with all relevant guidelines and regulations. The TriNetX Research Network complies with the HIPAA Privacy Rule under 45 CFR §164.514(b)(1) through expert-determined de-identification standards, and all data were de-identified before analysis. The study did not involve human subjects research as defined under 45 CFR §46.102 because the study team accessed only preexisting de-identified data and did not access protected health information, interact with individual subjects, or obtain identifiable private information. Therefore, institutional review board approval was not required. Because the study involved only secondary analysis of de-identified data, informed consent from subjects or their legal guardians was not required.

In accordance with TriNetX privacy rules, event counts of fewer than 10 patients are displayed as “ ≤ 10” to protect patient confidentiality. Statistical estimates including risk ratios and confidence intervals for these outcomes are computed by the TriNetX platform using the actual underlying counts prior to obfuscation and are reported as calculated.

## Results

### Cohort sizes and matching

Each of the four exposure cohorts was successfully balanced using one-to-one propensity score matching to create comparable cohorts for analysis. The analytic sample included 2421 cannabis-only users, 36,966 nicotine-only users, and 2338 concurrent users, each matched to non-user or cannabis-only comparators. Baseline demographic and clinical characteristics were well balanced across all matched cohorts, with standardized mean differences below 0.10 for every covariate. These balanced cohorts formed the basis for all subsequent outcome comparisons. Results of pre and post matching can be found in Table 1. Across comparisons of cannabis-only users and combined cannabis and nicotine users, baseline characteristics including age, sex, race, fracture type, and comorbidities were balanced with standardized mean differences below 0.10, confirming successful covariate matching. However, covariate balance was not fully achieved for HbA1c in the cannabis-only cohort (SMD = 0.146) and concurrent user cohort (SMD = 0.102), despite each sub-category being well balanced (Supplemental Fig. 1). All trauma-specific covariates, including fracture location, open versus closed fracture status, type of fixation procedure, polytrauma, and preoperative antibiotic administration, were included in matching and achieved standardized mean differences below 0.10 in all cohorts.

### Cannabis-only users

Cannabis-only users experienced higher rates of several postoperative complications compared with matched non-users in this cohort. Specific values and results of these outcome analyses can be found in Table 2. Surgical complications were increased in cannabis-only users compared with non-users, including deep implant infection (RD: 1.467%; RR: 2.260; 95% CI: 1.453–3.515), nonunion or malunion (RD: 2.138%; RR: 1.462; 95% CI: 1.153–1.853), and reoperation (RD: 3.387%; RR: 1.299; 95% CI: 1.122–1.505). Nerve palsy was also more frequent among cannabis-only users (RD: 0.644%; RR: 2.171; 95% CI: 1.127–4.181), although this finding was considered exploratory given the absence of a clear mechanistic basis. No significant differences were observed for wound dehiscence (RD: 0.416%; RR: 1.774; 95% CI: 0.901–3.495), superficial wound infection (RD: 0.413%; RR: 1.144; 95% CI: 0.850–1.539), nosocomial infection (RD: 0%; RR: 1.000; 95% CI: 0.417–2.415), irrigation and debridement (RD: 0.337%; RR: 1.805; 95% CI: 0.835–3.901), amputation (RD: 0%; RR: 1.000; 95% CI: 0.417–2.398), and DVT (RD: 0%; RR: 1.000; 95% CI: 0.417–2.384).

**Table 2:** Outcomes of lower extremity surgical fixation in cannabis only users, nicotine only users, and concurrent users.

Metric	Cannabis-only users vs non users							Nicotine-only users vs non-users							Concurrent users vs cannabis-only users						
	Cannabis only users with outcome	Non-users with outcome	RD (%)	95% CI (%)	*P*-value	RR	95% CI	Nicotine only users with outcome	Non-users with outcome	RD (%)	95% CI (%)	*P*-value	RR	95% CI	Concurrent users with outcome	Cannabis only users with outcome	RD (%)	95% CI (%)	*P*-value	RR	95% CI
Wound Dehiscence	23	13	0.416	(− 0.069, 0.902)	0.0926	1.774	(0.901, 3.495)	459	262	0.536	(0.394, 0.679)	** < 0.0001**	1.754	(1.508, 2.04)	28	21	0.303	(− 0.284, 0.89)	0.3118	1.336	(0.761, 2.345)
Superficial Wound Infection	89	79	0.413	(− 0.619, 1.639)	0.3759	1.144	(0.85, 1.539)	1844	1319	1.745	(1.413, 2.076)	** < 0.0001**	1.435	(1.34, 1.538)	137	85	2.823	(1.438, 4.209)	** < 0.0001**	1.703	(1.309, 2.217)
Deep Implant Infection	63	28	1.467	(0.696, 2.238)	**0.0002**	2.26	(1.453, 3.515)	1172	769	1.114	(0.881, 1.347)	** < 0.0001**	1.531	(1.40, 1.675)	97	56	1.795	(0.763, 2.827)	**0.0007**	1.742	(1.26, 2.408)
Nonunion or Malunion	158	109	2.138	(0.812, 3.464)	**0.0016**	1.462	(1.153, 1.853)	1296	916	1.063	(0.812, 1.313)	** < 0.0001**	1.421	(1.308, 1.545)	107	82	1.133	(− 0.021, 2.288)	0.0542	1.317	(0.994, 1.746)
Nerve Palsy	28	13	0.644	(0.113, 1.175)	**0.0174**	2.171	(1.127, 4.181)	362	281	0.241	(0.101, 0.382)	**0.0008**	1.304	(1.116, 1.523)	23	27	− 0.162	(− 0.775, 0.451)	0.6043	0.864	(0.497, 1.502)
Irrigation and Debridement	18	≤ 10*	0.337	(− 0.097, 0.772)	0.1278	1.805	(0.835, 3.901)	250	190	0.172	(0.058, 0.285)	**0.003**	1.328	(1.10, 1.603)	25	19	0.282	(− 0.288, 0.851)	0.3318	1.34	(0.74, 2.427)
Amputation	≤ 10*	≤ 10*	0	(− 0.364, 0.363)	1	1	(0.417, 2.398)	17	≤ 10*	0.019	(− 0.009, 0.047)	0.1772	1.701	(0.779, 3.715)	≤ 10*	0	0.428	(0.163, 0.693)	**0.0015**	–	(− , −)
Reoperation	356	274	3.387	(1.494, 5.28)	0.0005	1.299	(1.122, 1.505)	4824	3903	2.491	(2.027, 2.956)	** < 0.0001**	1.236	(1.188, 1.286)	432	335	4.149	(2.029, 6.268)	**0.0001**	1.29	(1.132, 1.47)
DVT	≤ 10*	≤ 10*	0	(− 0.364, 0.363)	0.999	1	(0.417, 2.384)	119	100	0.052	(− 0.027, 0.13)	0.1998	1.189	(0.912, 1.551)	≤ 10*	≤ 10*	0	(− 0.377, 0.376)	0.9985	0.999	(0.417, 2.396)
PE	40	33	0.289	(− 0.397, 0.976)	0.4091	1.212	(0.767, 1.915)	699	647	0.141	(− 0.052, 0.333)	0.1526	1.08	(0.972, 1.201)	39	39	0	(− 0.734, 0.734)	1	1	(0.644, 1.553)
Transfusions	77	46	1.386	(0.46, 2.313)	**0.0033**	1.705	(1.189, 2.446)	1264	1009	0.751	(0.49, 1.013)	** < 0.0001**	1.273	(1.164, 1.370)	94	69	1.15	(0.043, 2.258)	**0.0417**	1.371	(1.01, 1.86)
Pneumonia	22	26	− 0.175	(− 0.759, 0.408)	0.556	0.844	(0.48, 1.485)	897	727	0.508	(0.277, 0.74)	** < 0.0001**	1.236	(1.122, 1.361)	36	20	0.753	(0.092, 1.414)	**0.0252**	1.842	(1.07, 3.172)
Nosocomial Infection	≤ 10*	≤ 10*	0	(− 0.361, 0.364)	0.9941	1	(0.417, 2.415)	123	79	0.12	(0.044, 0.196)	**0.0019**	1.559	(1.176, 2.067)	≤ 10*	≤ 10*	0	(− 0.375, 0.376)	0.999	1	(0.417, 2.399)
Respiratory Failure	22	24	− 0.071	(− 0.661, 0.52)	0.8138	0.933	(0.525, 1.659)	897	855	0.171	(− 0.074, 0.416)	0.1706	1.067	(0.973, 1.17)	38	20	0.876	(0.177, 1.576)	**0.0138**	1.942	(1.136, 3.378)
ARDS	≤ 10*	≤ 10*	0	(− 0.361, 0.361)	1	1	(0.417, 2.398)	179	147	0.087	(− 0.009, 0.182)	0.0757	1.218	(0.979, 1.514)	≤ 10*	≤ 10*	0	(− 0.374, 0.374)	1	1	(0.417, 2.398)
Myocardial Infarction	13	≤ 10*	0.13	(− 0.263, 0.524)	0.5162	1.312	(0.576, 2.986)	392	339	0.154	(0.005, 0.302)	**0.0424**	1.161	(1.005, 1.342)	≤ 10*	12	− 0.086	(− 0.487, 0.315)	0.6758	0.837	(0.362, 1.933)
Acute Kidney Injury	30	28	0.124	(− 0.537, 0.785)	0.7126	1.101	(0.66, 1.836)	818	846	− 0.056	(− 0.297, 0.185)	0.648	0.978	(0.89, 1.075)	42	28	0.716	(− 0.057, 1.49)	0.0688	1.548	(0.963, 2.487)
Stroke	13	≤ 10*	0.129	(− 0.265, 0.522)	0.5221	1.307	(0.573, 2.991)	262	274	− 0.031	(− 0.158, 0.096)	0.6337	0.96	(0.811, 1.136)	≤ 10*	12	− 0.087	(− 0.487, 0.313)	0.6706	0.834	(0.361, 1.927)
Death	44	46	− 0.082	(− 0.844, 0.68)	0.833	0.957	(0.635, 1.441)	1828	1538	0.797	(0.493, 1.10)	** < 0.0001**	1.19	(1.113, 1.271)	41	42	− 0.043	(− 0.801, 0.715)	0.9118	0.976	(0.637, 1.495)
Anxiety	88	49	2.621	(1.437, 3.805)	** < 0.0001**	2.145	(1.521, 3.024)	1351	1055	1.472	(1.143, 1.802)	** < 0.0001**	1.424	(1.316, 1.541)	109	84	1.885	(0.298, 3.473)	**0.0194**	1.388	(1.053, 1.83)
Depression	66	35	1.522	(0.648, 2.397)	**0.0006**	2.009	(1.339, 3.014)	894	627	0.901	(0.674, 1.127)	** < 0.0001**	1.492	(1.348, 1.65)	72	63	0.54	(− 0.546, 1.626)	0.3288	1.18	(0.846, 1.646)
Opioid use	18	≤ 10*	0.345	(− 0.089, 0.778)	0.1183	1.831	(0.847, 3.959)	306	91	0.602	(0.494, 0.709)	** < 0.0001**	3.431	(2.716, 4.333)	37	17	0.972	(0.321, 1.622)	**0.0031**	2.31	(1.304, 4.089)
Chronic Pain	103	95	0.63	(− 0.606, 1.867)	0.3169	1.149	(0.875, 1.509)	2086	1451	2.385	(2.021, 2.749)	** < 0.0001**	1.53	(1.434, 1.633)	149	100	2.678	(1.184, 4.171)	**0.0004**	1.549	(1.211, 1.98)
Readmission	183	141	1.735	(0.328, 3.142)	**0.0157**	1.298	(1.05, 1.605)	2260	1502	2.051	(1.734, 2.367)	** < 0.0001**	1.505	(1.412, 1.604)	281	172	4.662	(2.972, 6.352)	** < 0.0001**	1.634	(1.363, 1.958)

Definition is a p value <0.05.

Among medical complications, cannabis-only users had higher transfusion rates (RD: 1.386%; RR: 1.705; 95% CI: 1.189–2.446). No significant differences were seen for PE (RD: 0.289%; RR: 1.212; 95% CI: 0.767–1.915), pneumonia (RD: –0.175%; RR: 0.844; 95% CI: 0.480–1.485), respiratory failure (RD: –0.071%; RR: 0.933; 95% CI: 0.525–1.659), ARDS (RD: 0%; RR: 1.000; 95% CI: 0.417–2.398), myocardial infarction (RD: 0.130%; RR: 1.312; 95% CI: 0.576–2.986), acute kidney injury (RD: 0.124%; RR: 1.101; 95% CI: 0.660–1.836), stroke (RD: 0.129%; RR: 1.307; 95% CI: 0.573–2.991), and death (RD: –0.082%; RR: 0.957; 95% CI: 0.635–1.441). Coagulation parameters were not significantly different (PTT: 35.478 vs 34.809 s, *P* = 0.6097; PT: 13.585 vs 14.213 s, *P* = 0.0976).

**Fig. 2:**
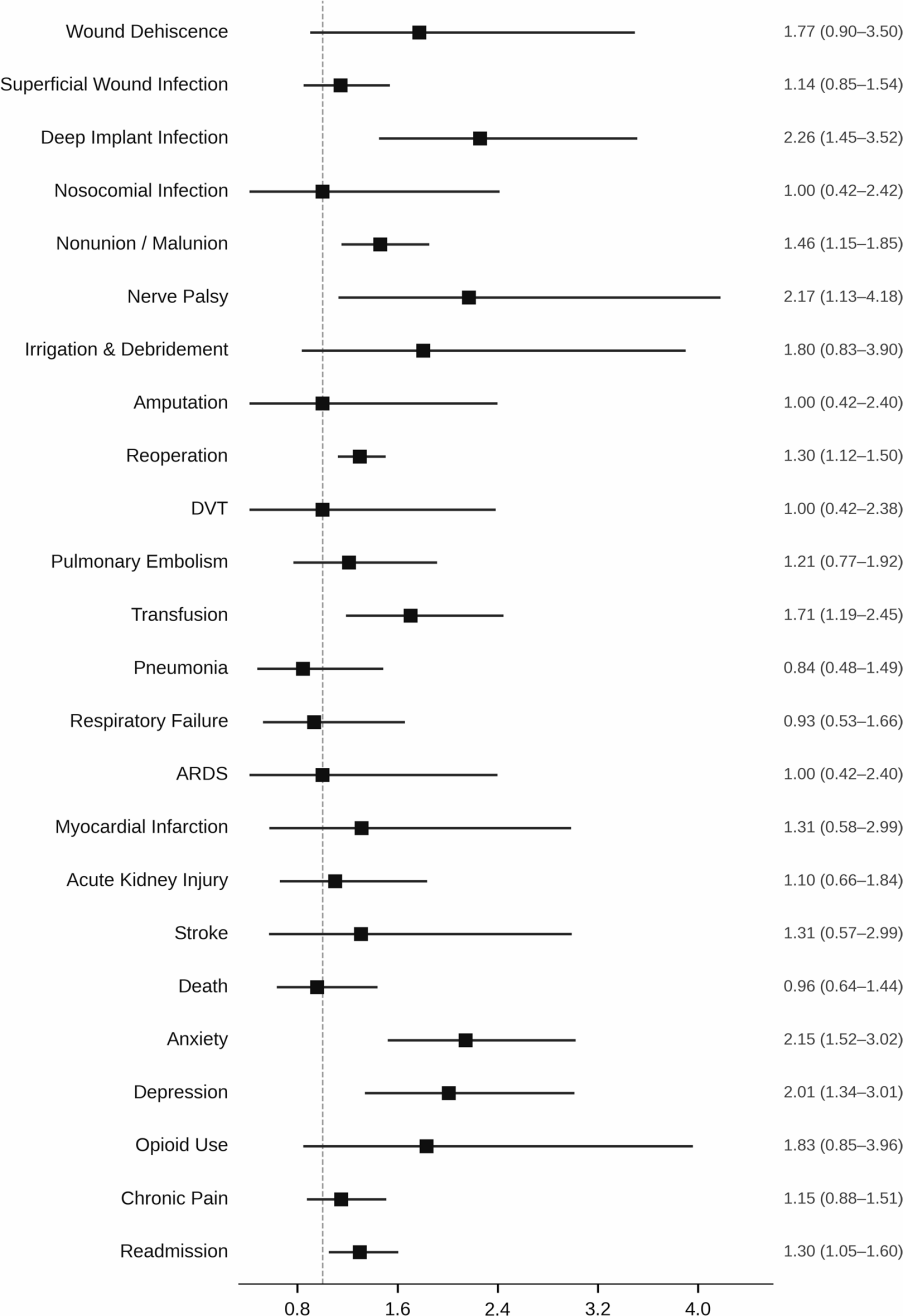
Cannabis-Only Users Versus Matched Non-Users: Postoperative Complication Risk Profile: Forest plot of risk ratios (RRs) and 95% confidence intervals for postoperative outcomes among cannabis-only users compared with matched non-users (n = 2421 per group). Numerical RR and 95% confidence interval values are displayed alongside each outcome. The vertical reference line indicates RR = 1.0. Exact event counts are provided in Table 2.

**Fig. 3:**
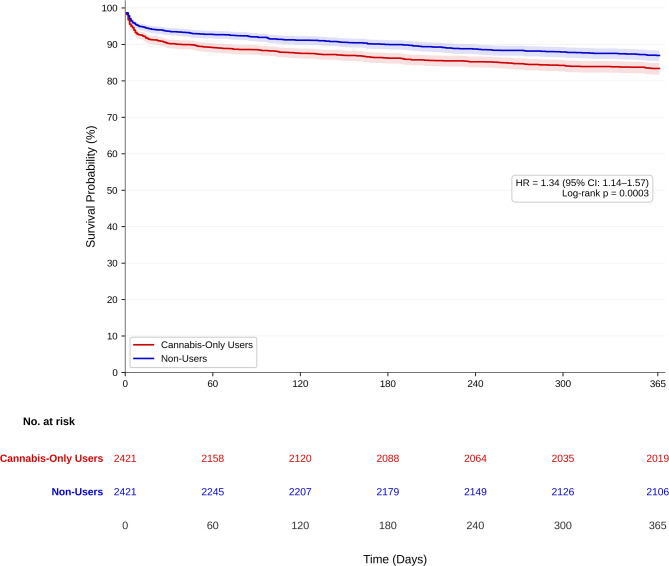
Cannabis-Only Users Versus Matched Non-Users: 1-Year Reoperation-Free Survival Curve: Kaplan–Meier curve for 1-year reoperation-free survival among cannabis-only users compared with matched non-users. The figure includes the number at risk over time, hazard ratio with 95% confidence interval, and log-rank p-value.

Psychosocial complications were also more common, including anxiety (RD: 2.621%; RR: 2.145; 95% CI: 1.521–3.024), depression (RD: 1.522%; RR: 2.009; 95% CI: 1.339–3.014), and readmission (RD: 1.735%; RR: 1.298; 95% CI: 1.050–1.605). No significant differences were observed for opioid use (RD: 0.345%; RR: 1.831; 95% CI: 0.847–3.959) or chronic pain (RD: 0.630%; RR: 1.149; 95% CI: 0.875–1.509). Comparative risk ratios for outcomes can be found in Fig. 2. Survival curves for reoperation for cannabis only users can be found in Fig. 3.

### Nicotine only users

Nicotine-only users demonstrated elevated rates of surgical, medical, and psychosocial complications relative to matched non-users. Specific values and results of these outcome analyses can be found in Table 2. Surgical risks were consistently higher in nicotine-only users, including wound dehiscence (RD: 0.536%; RR: 1.754; 95% CI: 1.508–2.040), superficial wound infection (RD: 1.745%; RR: 1.435; 95% CI: 1.340–1.538), deep implant infection (RD: 1.114%; RR: 1.531; 95% CI: 1.400–1.675), nosocomial infection (RD: 0.120%; RR: 1.559; 95% CI: 1.176–2.067), nonunion or malunion (RD: 1.063%; RR: 1.421; 95% CI: 1.308–1.545), nerve palsy (RD: 0.241%; RR: 1.304; 95% CI: 1.116–1.523), irrigation and debridement (RD: 0.172%; RR: 1.328; 95% CI: 1.100–1.603), and reoperation (RD: 2.491%; RR: 1.236; 95% CI: 1.188–1.286). No significant differences were observed for amputation (RD: 0.019%; RR: 1.701; 95% CI: 0.779–3.715), DVT (RD: 0.052%; RR: 1.189; 95% CI: 0.912–1.551), or PE (RD: 0.141%; RR: 1.080; 95% CI: 0.972–1.201).

Medical complications with significantly higher rates among nicotine only users included transfusion (RD: 0.751%; RR: 1.273; 95% CI: 1.164–1.370), pneumonia (RD: 0.508%; RR: 1.236; 95% CI: 1.122–1.361), myocardial infarction (RD: 0.154%; RR: 1.161; 95% CI: 1.005–1.342), and death (RD: 0.797%; RR: 1.190; 95% CI: 1.113–1.271). No significant differences were found for respiratory failure (RD: 0.171%; RR: 1.067; 95% CI: 0.973–1.170), ARDS (RD: 0.087%; RR: 1.218; 95% CI: 0.979–1.514), acute kidney injury (RD: –0.056%; RR: 0.978; 95% CI: 0.890–1.075), or stroke (RD: –0.031%; RR: 0.960; 95% CI: 0.811–1.136). Coagulation parameters showed a significantly shorter PT among nicotine only users (14.026 vs 14.750 s; *P* < 0.0001) and no significant difference in PTT (35.202 vs 35.362 s; *P* = 0.6112) (Table 3).

**Table 3:** Comparative analysis of coagulation profiles among cannabis only users, nicotine only users, and concurrent users.

Outcome	Comparison	Mean (Group 1)	SD (Group 1)	Mean (Group 2)	SD (Group 2)	t	*P*-value
Partial thromboplastin time (PTT)	Cannabis-only users vs non-users	35.478	16.5	34.809	12.883	0.511	0.6097
	Nicotine-only users vs non-users	35.202	15.58	35.362	15.471	− 0.508	0.6112
	Concurrent users vs cannabis only users	34.484	15.091	35.612	17.867	− 0.837	0.4029
Prothrombin time (PT)	Cannabis-only users vs non-users	13.585	3.663	14.213	6.477	− 1.659	0.0976
	Nicotine-only users vs non-users	14.026	5.579	14.75	6.845	− 6.723	** < 0.0001**
	Concurrent users vs cannabis-only users	13.758	5.085	13.604	3.519	0.512	0.6091

Definition is a p value <0.05.

**Fig. 4:**
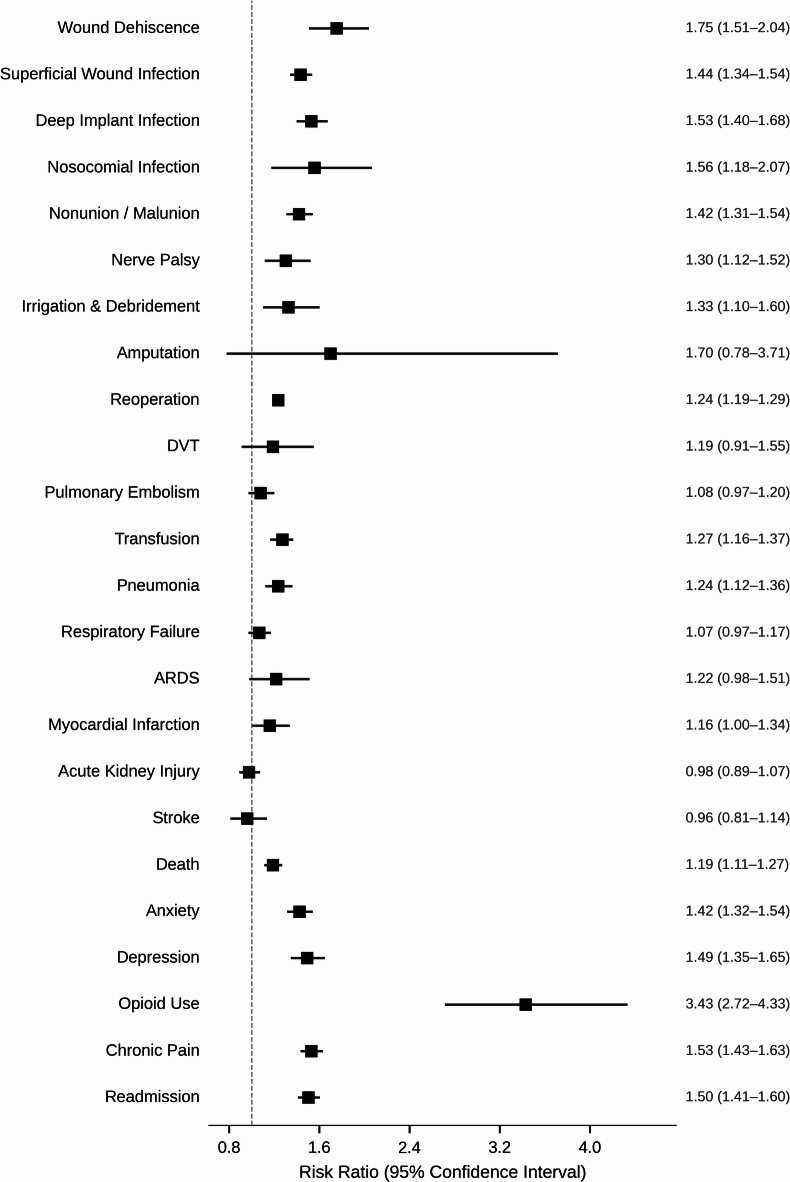
Nicotine-only users versus matched non-users: postoperative complication risk profile: forest plot of risk ratios (RRs) and 95% confidence intervals for postoperative outcomes among nicotine-only users compared with matched non-users (n = 36,966 per group). Numerical RR and 95% confidence interval values are displayed alongside each outcome. The vertical reference line indicates RR = 1.0. Exact event counts are provided in Table 2.

**Fig. 5:**
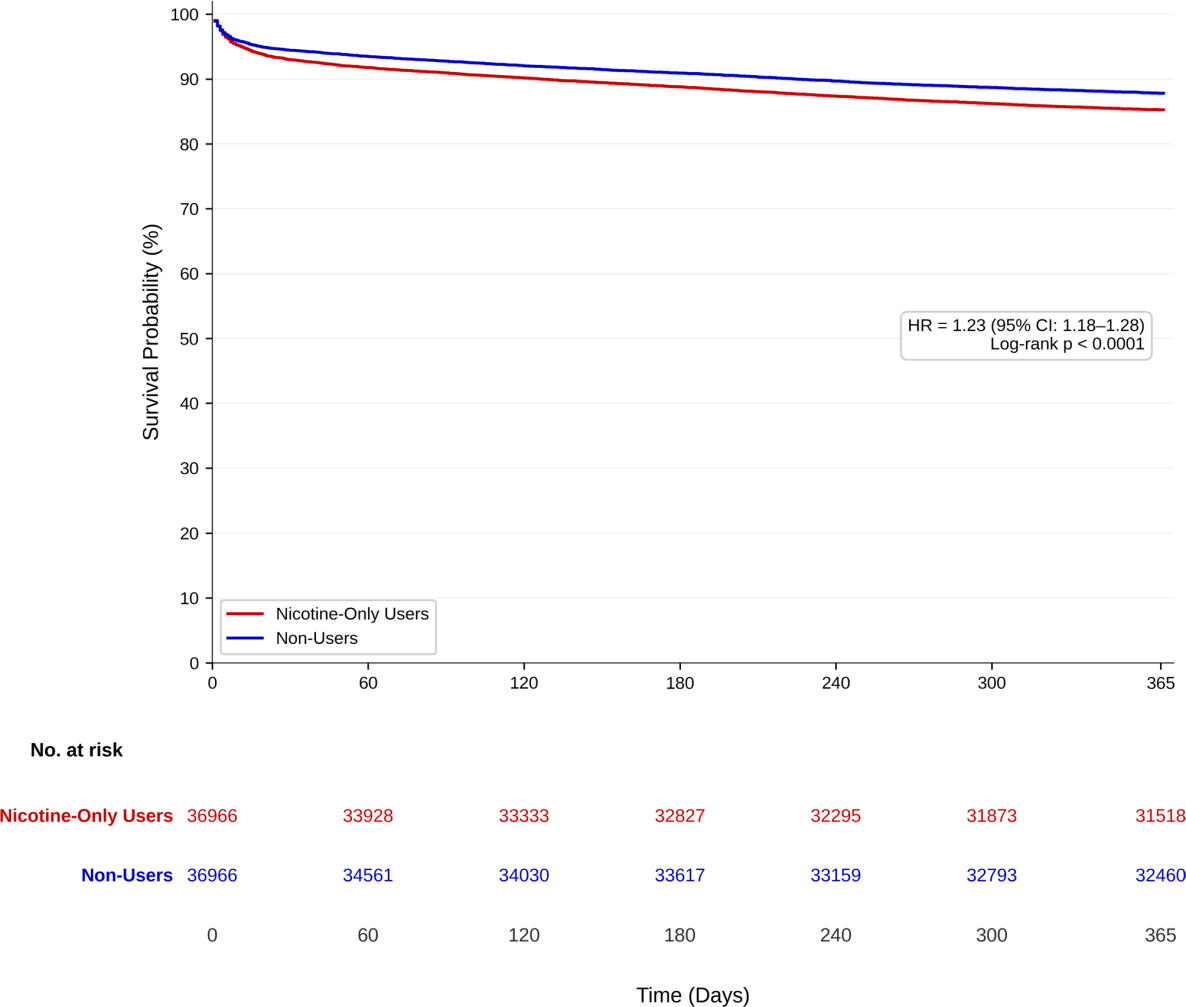
Nicotine-Only Users Versus Matched Non-Users: 1-Year Reoperation-Free Survival Curve: Kaplan–Meier curve for 1-year reoperation-free survival among nicotine-only users compared with matched non-users. The figure includes the number at risk over time, hazard ratio with 95% confidence interval, and log-rank *p*-value.

Psychosocial morbidity was also greater among nicotine-only users, including anxiety (RD: 1.472%; RR: 1.424; 95% CI: 1.316–1.541), depression (RD: 0.901%; RR: 1.492; 95% CI: 1.348–1.650), opioid use (RD: 0.602%; RR: 3.431; 95% CI: 2.716–4.333), chronic pain (RD: 2.385%; RR: 1.530; 95% CI: 1.434–1.633), and readmission (RD: 2.051%; RR: 1.505; 95% CI: 1.412–1.604). Comparative risk ratios for outcomes can be found in Fig. 4. Survival curves for reoperation for cannabis only users can be found in Fig. 5.

### Concurrent users

Concurrent cannabis-and-nicotine users had higher postoperative risk compared with cannabis-only users. Specific values and results of these outcome analyses can be found in Table 2. Surgical complications were notably higher, including superficial wound infection (RD: 2.823%; RR: 1.703; 95% CI: 1.309–2.217), deep implant infection (RD: 1.795%; RR: 1.742; 95% CI: 1.260–2.408), amputation (RD: 0.428%; RR not estimable), and reoperation (RD: 4.149%; RR: 1.290; 95% CI: 1.132–1.470). No significant differences were observed for wound dehiscence (RD: 0.303%; RR: 1.336; 95% CI: 0.761–2.345), nosocomial infection (RD: 0%; RR: 1.000; 95% CI: 0.417–2.399), nonunion or malunion (RD: 1.133%; RR: 1.317; 95% CI: 0.994–1.746), nerve palsy (RD: –0.162%; RR: 0.864; 95% CI: 0.497–1.502), irrigation and debridement (RD: 0.282%; RR: 1.340; 95% CI: 0.740–2.427), DVT (RD: 0%; RR: 0.999; 95% CI: 0.417–2.396), and PE (RD: 0%; RR: 1.000; 95% CI: 0.644–1.553).

Among medical complications, concurrent users had higher rates of transfusion (RD: 1.150%; RR: 1.371; 95% CI: 1.010–1.860), pneumonia (RD: 0.753%; RR: 1.842; 95% CI: 1.070–3.172), and respiratory failure (RD: 0.876%; RR: 1.942; 95% CI: 1.136–3.378). No significant differences were observed for ARDS (RD: 0%; RR: 1.000; 95% CI: 0.417–2.398), myocardial infarction (RD: –0.086%; RR: 0.837; 95% CI: 0.362–1.933), acute kidney injury (RD: 0.716%; RR: 1.548; 95% CI: 0.963–2.487), stroke (RD: –0.087%; RR: 0.834; 95% CI: 0.361–1.927), or death (RD: –0.043%; RR: 0.976; 95% CI: 0.637–1.495).

**Fig. 6:**
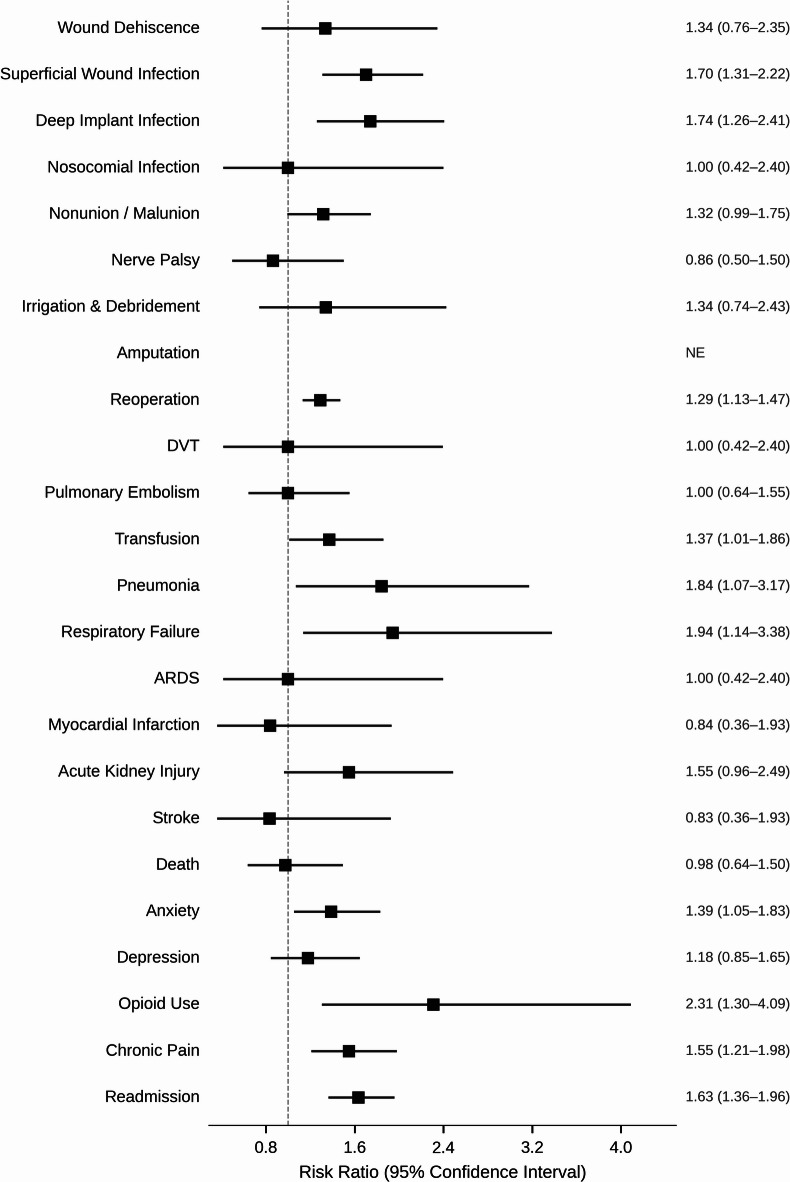
Concurrent Cannabis-and-Nicotine Users Versus Matched Cannabis-Only Users: Postoperative Complication Risk Profile: Forest plot of risk ratios (RRs) and 95% confidence intervals for postoperative outcomes among concurrent cannabis-and-nicotine users compared with matched cannabis-only users (n = 2338 per group). Numerical RR and 95% confidence interval values are displayed alongside each outcome. The vertical reference line indicates RR = 1.0. Exact event counts are provided in Table 2.

**Fig. 7:**
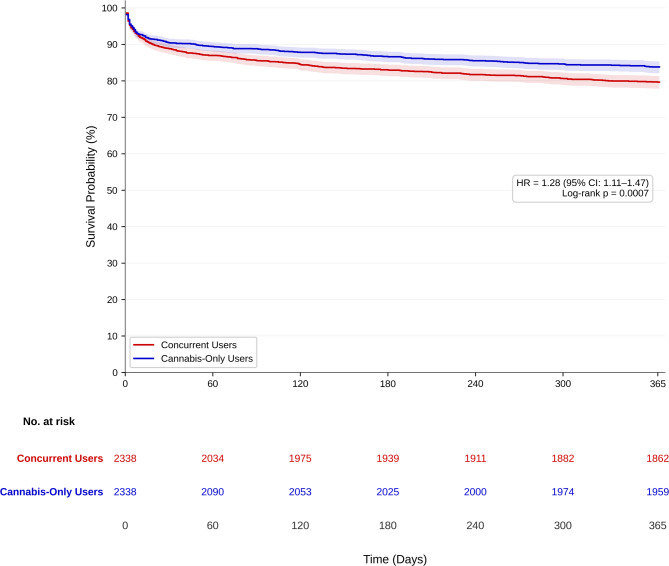
Concurrent Users Versus Matched Cannabis-Only Users: 1-Year Reoperation-Free Survival Curve: Kaplan–Meier curve for 1-year reoperation-free survival among concurrent cannabis-and-nicotine users compared with matched cannabis-only users. The figure includes the number at risk over time, hazard ratio with 95% confidence interval, and log-rank p-value.

Psychosocial complications were also elevated, including anxiety (RD: 1.885%; RR: 1.388; 95% CI: 1.053–1.830), opioid use (RD: 0.972%; RR: 2.310; 95% CI: 1.304–4.089), chronic pain (RD: 2.678%; RR: 1.549; 95% CI: 1.211–1.980), and readmission (RD: 4.662%; RR: 1.634; 95% CI: 1.363–1.958). No significant differences were observed for depression (RD: 0.540%; RR: 1.180; 95% CI: 0.846–1.646). Coagulation parameters were not significantly different (PTT: 34.484 vs 35.612 s, *P* = 0.4029; PT: 13.758 vs 13.604 s, *P* = 0.6091). Comparative risk ratios for outcomes can be found in Fig. 6. Survival curves for reoperation for cannabis only users can be found in Fig. 7.

### Sensitivity analysis for cannabis exposure ascertainment

A sensitivity analysis was performed to assess ascertainment of cannabis exposure in the TriNetX database. According to the National Survey on Drug Use and Health (NSDUH), approximately 6.8% of Americans age 12 or older report having a cannabis use disorder. Applying this prevalence to more than 150 million patients in the TriNetX Research database network would predict roughly 10.2 million cannabis users. However, only 1,666,438 patients (1.11%) in the database carried an ICD-10-CM diagnosis code for cannabis-related disorders. This discrepancy suggests substantial underascertainment of cannabis exposure in structured electronic health record diagnosis fields.

### Sensitivity analysis for unmeasured confounding

E-values were calculated for all statistically significant associations to assess vulnerability to unmeasured confounding (Supplemental Table 2). Among cannabis-only users, E-values ranged from 1.92 (reoperation, readmission) to 3.95 (deep implant infection). Among nicotine-only users, E-values ranged from 1.59 (myocardial infarction) to 6.32 (opioid use). Among concurrent users, E-values ranged from 1.90 (reoperation) to 4.05 (opioid use). The majority of significant associations had E-values exceeding 2.0, indicating that a substantial degree of unmeasured confounding would be required to fully explain the observed effects. Full E-value measurements can be found in Supplemental Table 2.

## Discussion

In this large retrospective cohort, cannabis use was associated with higher rates of surgical and psychosocial complications after orthopedic trauma surgery. Nicotine use was linked to a broader range of adverse outcomes, and concurrent exposure was associated with compounded risk. These findings indicate that documented cannabis and nicotine use may identify patients at increased risk for postoperative morbidity in surgical populations.

These findings are consistent with emerging evidence that cannabis exposure may adversely influence perioperative outcomes through immune, vascular, and metabolic mechanisms. Experimental data show that cannabinoids modulate immune and inflammatory responses by suppressing cytokine signaling, promoting regulatory immune phenotypes, and reducing monocyte-derived interleukin-1β production^13,14^. Such effects may weaken host defenses, increasing susceptibility to infection and delayed tissue repair.

The increased rates of nonunion and malunion among cannabis users may reflect dose-dependent effects on bone remodeling; however, this mechanistic interpretation remains speculative because TriNetX does not capture cannabis dose, frequency, route, chronicity, or recency of exposure. Prior experimental studies suggest that low cannabinoid concentrations may promote osteoblast differentiation, whereas higher concentrations may inhibit bone formation and enhance osteoclast activity, resulting in a biphasic response^15,16^. Because exposure intensity could not be quantified in this cohort, these biological mechanisms should be interpreted as plausibility arguments rather than direct evidence of causation. In addition to dose-related effects, age may influence the relationship between cannabis use and outcomes, with some literature indicating more frequent medical complications in older populations undergoing procedures such as arthroplasty and fragility fracture fixation, compared with procedures performed for high energy trauma in younger patients^8–12^.

Although we observed an association between cannabis use and increased rates of nerve palsy, this finding should be considered exploratory. There is currently no high-quality evidence directly linking cannabis exposure to peripheral nerve injury, and consensus guidelines do not recognize neuropathy or nerve damage as established complications of cannabis use^17^. This association may reflect coding artifact, differences in injury pattern, surgical positioning, or residual confounding rather than a causal effect of cannabis exposure. Further studies with procedure-level detail, laterality, neurologic examination findings, and adjudicated nerve injury outcomes are needed to determine whether this association is reproducible.

Nicotine’s known vasoconstrictive and anti-osteogenic effects likely contribute to its broader and more pronounced association with adverse surgical and medical outcomes. The consistency of these findings with prior literature also supports the validity of the analytic approach and strengthens the observed cannabis related signals^18,19^. Its inclusion in this study also serves as an internal control, reinforcing the validity of our methodology. The higher complication rates observed among concurrent cannabis-and-nicotine users suggest that combined exposure may compound perioperative risk. Prior work has identified similarly elevated complication rates among patients with combined cannabis and tobacco exposure after total joint arthroplasty and ankle fracture fixation^20,21^. While both substances may affect immune cell recruitment and resolution of inflammation, their combined influence may plausibly worsen wound healing through overlapping mechanisms.

No increase in venous thromboembolism was observed, although prior studies have reported conflicting results. Cannabis exposure has been linked to platelet activation and endothelial dysfunction, which may promote a prothrombotic state even when standard coagulation assays appear normal^22,23^. Consistent with this possibility, LoPolito et al. found prolonged thromboelastography (TEG) reaction times among trauma patients with cannabis exposure, suggesting delayed clot initiation^24^. These findings indicate that advanced coagulation assays may better detect subtle cannabis-related changes and help refine perioperative risk stratification.

Cannabis, nicotine, and concurrent use were each associated with higher rates of anxiety, depression, chronic pain, and readmission. These findings underscore the psychosocial dimension of substance use in surgical recovery and the importance of multidisciplinary perioperative care that includes mental-health and substance-use assessment. Although some literature suggests cannabis may reduce opioid requirements, we observed no protective effect, and concurrent use was associated with greater opioid utilization. Comprehensive perioperative screening and counseling integrating pain management, mental health, and substance use evaluation are warranted. This approach is consistent with ASRA Pain Medicine consensus guidelines, which recommend universal preoperative screening for cannabis use, including assessment of product type, route of administration, timing of last use, amount, and frequency^17^.

## Limitations

This study has several limitations inherent to retrospective analyses of electronic health record data. Cannabis and nicotine exposure classification relied on ICD-10-CM diagnosis codes rather than direct measures of substance use behavior. These codes may undercapture true exposure because of inconsistent screening, patient nondisclosure, stigma, and institutional coding variation, which could bias results toward the null. Therefore, the cannabis cohort likely represents patients with documented cannabis-related diagnoses rather than all patients who use cannabis. The database does not capture cannabis dose, frequency, route, or recency of use, and detailed socioeconomic or behavioral variables were unavailable. Although residual confounding is possible, rigorous propensity score matching and adjustment for major comorbidities, fracture characteristics, and polysubstance use strengthen the validity of our findings. Because this study evaluated multiple outcomes across three cohort comparisons, the use of a nominal *P* < 0.05 threshold without formal multiplicity adjustment increases the possibility of type I error. Statistically significant findings, particularly borderline associations, should therefore be interpreted cautiously. Although 1:1 propensity score matching generated matched cohorts, TriNetX performs post-matching analyses using independent-sample methods rather than paired or conditional approaches. Accordingly, paired t tests, conditional logistic regression, stratified Cox models, and IPTW could not be performed, and results should be interpreted as matched-cohort rather than conditional matched-pair analyses. Injury Severity Scores and time to antibiotic administration could not be directly assessed, but surrogate variables for polytrauma and preoperative antibiotic exposure were incorporated to mitigate these effects. Despite these limitations, the study’s large sample size and multi-institutional design provide an estimate of cannabis- and nicotine-associated perioperative risk.

## Conclusion

Cannabis use was associated with adverse perioperative outcomes after lower extremity fracture fixation. Using a large, multi-institutional electronic health record dataset, this study identified associations between cannabis and nicotine exposure and adverse surgical and psychosocial outcomes. These results support further prospective evaluation of standardized substance use screening and counseling strategies and highlight the need for prospective, dose response, and mechanistic investigations to guide clinical practice and public health policy.

## Supplementary Information

Below is the link to the electronic supplementary material.

Supplementary Material 1

Supplementary file1

## Data Availability

The data that support the findings of this study are derived from the TriNetX Research Network, a federated electronic health record platform that aggregates de-identified electronic health records from participating healthcare organizations. The data are accessible only to institutions with active TriNetX subscriptions and cannot be shared publicly due to licensing restrictions and patient privacy regulations. Researchers affiliated with member institutions can access the underlying data directly via the TriNetX Analytics platform to replicate or extend the analyses described in this study.
